# A multicenter study on surgical procedure selection and risk factor analysis of postoperative complications after TIP and Duckett hypospadias repair

**DOI:** 10.1186/s12894-022-01051-2

**Published:** 2022-08-25

**Authors:** YiWei Fang, Ning Sun, HongCheng Song, WeiPing Zhang, YunMan Tang, LuGang Huang, Yi Yang, Min Chao, Hong Ma, JingTi Zhang, XuHui Zhang, ShouLin Li, Ning Li, Chao Chen, DaWei He, WenBo Wu, Hua Xie, Yong Guan

**Affiliations:** 1 Department of Urology, National Center for Children’s Health, Beijing Children’s Hospital of Capital Medical University, Beijing, 100045 China; 2 Department of Pediatric Surgery, Department of Pediatric Surgery, Sichuan Academy of Medical Sciences – Sichuan Provincial People’s Hospital (SAMSPH), Chengdu, 610072 China; 3 Department of Pediatric Surgery, West China Hospital of Sichuan University, Chengdu, 610044 China; 4 Department of Pediatric Urology, Shengjing Hospital of China Medical University, Shenyang, 110004 China; 5 Department of Pediatric Urology, Anhui Children’s Hospital, Hefei, 230022 China; 6 Department of Pediatric Urology and General Thoracic Surgery, Affiliated Hospital of Zunyi Medical University, Zunyi, 563003 China; 7 Department of Urology, Xi’an Children’s Hospital, Xi’an, 710002 China; 8 Department of Urology, Shanxi Children’s Hospital, Taiyuan, 030006 China; 9 Department of Urology, Shenzhen Children’s Hospital, Shenzhen, 518034 China; 10 Department of Pediatric Urology, Tongji Hospital, Tongji Medical College, Huazhong University of Science & Technology, Wuhan, 230022 China; 11 Department of Pediatric Urology, The First Affiliated Hospital of Guangxi Medical University, Nanning, 530021 China; 12 Department of Urology, Children’s Hospital of Chongqing Medical University, Chongqing, 400015 China; 13 Department of Urology, Children’s Hospital of Jiangxi Province, Nanchang, 330006 China; 14 Department of Urology, Shanghai Children’s Hospital, Shanghai, 200062 China; 15 Department of Urology, Tianjin Children’s Hospital, Tianjin, 300134 China

**Keywords:** Proximal hypospadias, Tabularized incised plate, Ventral curvature, Urethroplasty

## Abstract

**Background:**

Hypospadias is a common congenital malformation in pediatric urology with surgery being the only curative treatment. Although there are hundreds of surgical methods for hypospadias, no single method can treat all types, and there are still high rates of postoperative complications. We performed this study to investigate surgical procedure selection and perform risk factor analysis of postoperative complications in hypospadias repair.

**Methods:**

Retrospective analysis was performed of complete clinical and follow-up data of children with hypospadias who were treated and followed up at 15 children’s clinical centers in Mainland China from December 2018 to December 2019. Children were divided into groups according to Barcat classification and surgical methods in order to analyze the surgical choice for different types of hypospadias and the influencing factors of different surgical methods for complications.

**Results:**

In total, 1011 patients were followed up for 26 months. According to Barcat classification, there were 248 cases of distal type hypospadias, 214 of intermediate, and 549 of proximal type. Transverse preputial island flap urethroplasty (Duckett) and tubularized incised plate urethroplasty (TIP) were performed in 375 (37.1%) and 336 cases (33.2%), respectively. The postoperative complication rate of distal hypospadias was 23.4% (15.8–57.1%), mid shaft 29.0% (22.7–40.0%), and proximal 43.7% (30.2–52.9%). Among the 375 patients in Duckett group, 192 had complications. Multivariate logistic analysis showed that the length of prepuce island flap (*OR* = 3.506, 95% *CI*: 2.258*–*5.442) was an independent risk factor for complications after Duckett operation (*P* < 0.001). In TIP group, there were 336 cases with 84 complications. Multivariate logistic analysis showed that the width of urethral plate after longitudinal resection (*OR* = 0.836, 95% *CI*: 0.742*–*0.942) and glans width (*OR* = 0.851, 95% *CI*: 0.749*–*0.965) were independent risk factors for postoperative complications after TIP (*P* = 0.003, *P* = 0.012).

**Conclusion:**

Several anatomical features play a role during the selection process among the different surgical approaches, including glans size, urethral plate width, and the meatal position. The width of the urethral plate and glans width were risk factors for postoperative complications after TIP. The length of prepuce island flap was a risk factor for complications after Duckett operation.

## Background

Hypospadias is one of the common congenital malformations in pediatric urology, with an incidence of 3.2/1000 [1]. Surgery is the only curative treatment for hypospadias. Although there are hundreds of surgical methods, no single method can treat all types of hypospadias, and there are still high rates of postoperative complications.

To improve the treatment of hypospadias, we carried out a multicenter hypospadias clinical research study, collecting and analyzing clinical and follow-up data of children with hypospadias following curative surgical intervention, focusing on surgical procedure selection and risk factors for postoperative complications in hypospadias.

## Methods

### Research subjects

The clinical data of children with hypospadias who were treated at 15 medical centers in mainland China from December 2018 to December 2019 were analyzed. The collection of patients was consecutive.

Inclusion criteria: initial diagnosis and treatment of hypospadias, as well as complete clinical and follow-up data. Specific clinical data was gathered, including: age, the position of urethral meatus, length of the penis, glans length and width, urethral defect length, urethral plate (UP) width, UP smoothness and elasticity, foreskin form, prepuce superficial vascular distribution, elasticity and smoothness of prepuce flap, urethroplasty, the degree of ventral curvature.

Exclusion criteria: hypospadias reoperation, incomplete clinical data, failure of follow-up as required, and disorders of sexual development.

Glans width was measured at the point of maximum width. UP width was defined the width of the UP by measuring widest distance between the lateral margins of the UP transversely. The flatness of UP and prepuce flap were divided into smooth, moderate and uneven. The elasticity of UP and prepuce flap were divided into good elasticity, general elasticity and poor elasticity. The degree of ventral curvature was objectively determined using a goniometer. Endoscopic cold light source was used to transilluminate the flap vascular pedicle and residual prepuce skin (during the operation). Prepuce superficial vascular distribution were divided into one blood vessel type, two blood vessels type, H-type, reticular type and no obvious type.

### Group

Grouping according to different urethroplasty methods: Meatal advancement and glanuloplasty incorporated procedure (MAGPI), tabularized incised plate urethroplasty (TIP), Onlay island flap urethroplasty, Mathieu, transverse preputial island flap urethroplasty (Duckett), Duckett combined with Duplay urethroplasty, Koyanagi, staged Duckett, and Byars flap.

According to Barcat classification of hypospadias: distal, middle, and proximal hypospadias.

### Follow-up

Patients were followed up 3-monthly for the first year, then annually and later biannually. The review in clinics was done by the consultants and trainees. Outcomes included urethral fistula, urethral stricture, diverticular dilatation, recurrent ventral curvature, residual chordee, and other complications requiring repeat surgical intervention.

### Statistical analysis

SPSS23.0 statistical software was used for data collection and analysis. Median and quartile spacing [*M*(*P*25, *P*75)] were used to describe the measurement data that did not obey normal distribution in univariate analysis. Non-parametric test (*Mann–Whitney U test*) was used to compare the data between groups. Non-parametric test (*Kruskal–Wallis H test*) was used to compare the measurement data of different types of hypospadias. Statistical data were described by number of cases and percentage, and chi-square test was used for comparison between groups. Select the single factor analysis of *P* < 0.05. The influencing factors were included in multivariate Logistic analysis. The Odds ratio (*OR*) and 95% Confidence Interval (*CI*) were calculated, and *P* < 0.05 was considered statistically significant.

## Results

### Subjects and clinical data

According to Barcat classification, there were 248 cases of distal type, 214 of middle type, and 549 of proximal type. The specific distribution of different types of hypospadias urethroplasty is shown in Table 1.

**Table 1:** Distribution and complications of different types of hypospadias urethroplasty

Classification of hypospadias	Operation	Number	Complications				Total
			Urethral fistula	Urethral stricture	Urethral diverticulum	Recurrent ventral curvature	
Distal	MAGPI	38	6				6
	TIP	172	22	17			39
	Onlay	31	9				9
	Mathieu	7	3	1			4
Middle	TIP	112	19	8	3		29
	Onlay	44	9		1		10
	Mathieu	8	3				3
	Duckett	50	18	7	7		20
Proximal	TIP	52	12	4			16
	Onlay	26	7	3			10
	Duckett	325	112	45	47	1	172
	Duckett + Duplay	30	3	9	3		12
	Koyanagi	53	10	7	1		16
	staged	63	6	6	2		14
Total		1011	239	107	64	1	360

The operative age was 7–180 months, with a median age of 28 months and a mean age of 35 months. Penis length 2.0–10.0 cm, mean 3.82 cm; glans length 5–22 mm, average 11.1 mm; glans width 6–25 mm, mean 13.6 mm; length of urethral defect 0.4–7.0 cm, average 2.7 cm. There were 140 cases of mild (< 15°), 298 cases of moderate (15–35°), and 573 cases of severe ventral curvature (> 35°). The clinical characteristics of different types of hypospadias are shown in Table 2.

**Table 2:** Clinical characteristics of different hypospadias classification

Classification of hypospadias	Number	Surgery age [month, M(P_25_,P_75_)]	Penis length [cm, M(P_25_,P_75_)]	Glans Length [mm, (P_25_,P_75_)]	Glans width [mm, M(P_25_,P_75_)]	Length of urethral defect [cm, M(P_25_,P_75_)]	Ventral curvature [°, (P_25_,P_75_)]
Distal	248	33(21,61.75)	4.0(3.5,5.0)	12(10,15)	15(13,16)	1.4(1.0,1.7)	30(15,40)
Middle	214	29.5(19,44)	3.55(3.18,4.3)	12(10,14)	14(12,15)	2.0(1.5,2.5)	30(20,48.75)
Proximal	549	26(19,36)	3.5(3.0,4.2)	10(8,12)	13(12,14)	3.5(3.0,4.0)	60(40,70)
*H*	*–*	28.333	45.829	85.021	74.163	627.504	259.928
*P*	*–*	0.000*	0.000*	0.000*	0.000*	0.000*	0.000*

Non-parametric test (*Kruskal–Wallis H test*) was used to compare the measurement data of different types of hypospadias

*Statistically significant

The age at the time of operation, penis length, glans length and width, urethral defect length, and penis ventral curvature had statistical significance in the different types of hypospadias (*P* < 0.001). There were statistically significant differences in glans width, urethral defect length, and penile curvature among the distal, middle and proximal groups (*P* < 0.001).

### Follow-up

All 1011 cases were appropriately followed up. The mean follow-up time was 26 months (24–36 months). The incidence of complications was 40.6% (411/1011), including urethral fistula in 239 cases (23.6%), urethral stricture in 107 cases (10.6%), diverticular dilatation in 64 cases (6.3%), and recurrence/residual ventral curvature in 1 case (0.09%). The incidence of complications involving the distal type was 23.4%, middle type 29.0%, and proximal type 43.7%. The specific incidence of postoperative complications of different types of hypospadias and different surgical procedures is shown in Table 1.

### Univariate and multivariate analysis of postoperative complications of TIP operation

In TIP group, 336 patients were 7–180 months old at the time of operation, with a median age of 29 months and an average age of 42 months. Penile length 2.0–10.0 cm, mean 4.1 cm; glans length 5–22 mm, mean 12.1 mm; glans width 7–25 mm, mean 14.5 mm; length of urethral defect 0.4–4.5 cm, average 1.8 cm. There were 124 cases of mild ventral curvature (< 15°), 191 cases of moderate ventral curvature (15–35°), and 21 cases of severe ventral curvature (> 35°). The flatness of UP was smooth in 268 cases, moderate in 59 cases, and uneven in 9 cases. The UP had good elasticity in 266 cases, moderate elasticity in 60 cases, and poor elasticity in 10 cases. The width of UP was 3–12 mm, average 5 mm, and the width of the UP after longitudinal incision was 6–18 mm, average 11 mm. There were 84 complications (25%, 84/336), as shown in Table 1.

Univariate analysis showed that the glans width, UP width, and the width of the UP after longitudinal incision (all *P* < 0.001) are risk factors for postoperative complications of TIP. There were no statistically significant differences in age at the time of operation, penis length, glans length, and urethral defect length between groups with and without complications (*P* > 0.05). The complications of the smooth urethral plate group were less than those of uneven urethral plate groups (23.1% and 32.4%, respectively), and those of the good urethral plate elasticity group were less than those of the poor urethral plate elasticity groups (23.3% and 31.4%, respectively), but the differences were not statistically significant (*P* > 0.05).

The glans width, UP width, and the width of the UP after longitudinal incision were analyzed using logistic analysis. It showed that the width of the UP after longitudinal incision (*OR* = 0.836, 95% *CI*: 0.742*–*0.942) and glans width (*OR* = 0.851, 95% *CI*: 0.749*–*0.965) were independent risk factors for postoperative complications after TIP surgery (*P* = 0.003, *P* = 0.012, respectively), as shown in Table 3.

**Table 3:** Logistic regression analysis of postoperative complications of TIP operation

	*β*	*SE*	*Wald*	*P*	*OR*	95% *CI*
Urethral plate width	− 0.050	0.094	0.281	0.596	0.951	0.791–1.144
Glans width	− 0.162	0.065	6.281	0.012*	0.851	0.749–0.965
Width of urethral plate after longitudinal incision	− 1.179	0.061	8.622	0.003*	0.836	0.742–0.942

Multivariate Logistic analysis. The Odds ratio (*OR*) and 95% Confidence Interval (*CI*) were calculated, and *P* < 0.05 was considered statistically significant

*Statistically significant

### Univariate and multivariate analysis of postoperative complications of Duckett operation

In the Duckett group, 375 patients were 8–134 months old, with a median age of 26 months and a mean age of 30 months. Penile length 2.0–6.8 cm, mean 3.7 cm; glans length 6–22 mm, average 11.2 mm; glans width 7–25 mm, mean 13.3 mm; length of urethral defect 1.7–6.0 cm, average 3.31 cm. There were 8 cases of mild ventral curvature (< 15°), 42 cases of moderate ventral curvature (15–35°), and 325 cases of severe ventral curvature (> 35°). Prepuce was monocular in 52 cases, binocular in 300 cases, and irregular in 23 cases. The prepuce superficial vascular distribution was one blood vessel type in 146 cases (38.9%), two blood vessel type in 140 cases (37.3%), H-type in 9 cases (2.4%), reticular type in 44 cases (11.7%), and no obvious type in 36 cases (9.6%). There were 188 cases of smooth prepuce island flaps, 144 cases of normal prepuce flaps, and 43 cases of uneven prepuce flaps. Good elasticity of prepuce island flaps in 193 cases, moderate elasticity in 162 cases, poor elasticity in 20 cases. The complication rate was 51.2% (192/375), as shown in Table 1.

Urethral defect length, prepuce island flap length, and ventral curvature (all *P* < 0.001) were risk factors for complications after Duckett operation. There were no statistically significant differences in age, penis length, glans length and width, prepuce morphology, and prepuce superficial vascular distribution between groups with and without complications (*P* > 0.05). Complications in the smooth flap group were less than those in moderate and uneven flap group (42.0% and 71.1%, respectively), and the difference between the two groups was statistically significant (*P* < 0.001). The complications in the group with good elasticity were less than those in the group with general elasticity and poor elasticity (40.9% and 62.1%, respectively), and the difference was statistically significant (*P* < 0.001), indicating that the elasticity and flatness of the prepuce flap were risk factors for postoperative complications after Duckett operation.

The length of prepuce island flap, ventral curvature, and the elasticity and flatness of prepuce flap were analyzed using multivariate logistic analysis. The results showed that length of the prepuce flap (*OR* = 3.506, 95% *CI*: 2.258*–*5.442) was an independent risk factor for postoperative complications after Duckett operation (*P* < 0.001) (Table 4). Thus, the risk of postoperative complications increases 3.506 times for each 1 cm increase in the length of the prepuce island flap.

**Table 4:** Logistic regression analysis of postoperative complications of Duckett operation

	*β*	*SE*	*Wald*	*P*	*OR*	95% *CI*
Prepuce island flap length	1.254	0.224	31.266	0.000*	3.506	2.258–5.442
Ventral curvature	0.003	0.005	0.284	0.594	1.003	0.993–1.013
Elasticity of prepuce flap	− 0.368	0.284	1.682	0.195	0.692	0.397–1.207
Flatness of prepuce flap	− 0.373	0.282	1.745	0.187	0.689	0.396–1.198

Multivariate Logistic analysis. The Odds ratio (*OR*) and 95% Confidence Interval (*CI*) were calculated, and *P* < 0.05 was considered statistically significant

*Statistically significant

## Discussion

### Current status of surgical options for hypospadias in China

The age at surgery for primary hypospadias repair is usually 6–18 months [2, 3]. We found that the average age of distal, middle, and proximal hypospadias at the time of operation was 33 months (21–61.75), 29.5 months (19–44), and 26 months (19–36), respectively, and that the difference was statistically significant, which may be related to the more severe deformity of proximal hypospadias, the heavier psychological burden on parents, and the earlier medical treatment.The penile growth rate is very slow in children three years and younger, and early treatment can reduce the psychological burden of this diagnosis on children, thus it is recommended to complete the operation within the first three years of life [4]. Age at surgery is not a risk factor for urethroplasty complications in pre-pubertal TIP urethroplasty repair [2]. We found that age at the time of pre-adolescent surgery was not a risk factor for postoperative complications of TIP and Duckett surgery (*P* = 0.460, *P* = 0.447*,* respectively), which was consistent with literature reports.

The more severe the degree of hypospadias, the worse the corresponding penile condition. In this multicenter study, we found that there were statistically significant differences in glans width, urethral defect length, and ventral curvature among the distal, middle, and proximal groups (*P* < 0.001). Therefore, in the selection of surgical methods, the penis conditions of children should be carefully considered.

The surgical treatment of hypospadias is mainly divided into three steps: correction of ventral curvature, urethroplasty, and penis appearance forming. Since TIP was first reported by Snodgrass in 1994, great progress has been made in the treatment of hypospadias in the recent 20 years. TIP is widely used in the treatment of distal hypospadias because the urethral opening can reach the glans with a cleft shape, resulting in a good penis appearance and a low incidence of complications such as urethral fistula and diverticulum [5]. We found that among 1011 children with hypospadias, there were 248 distal and 214 middle types. The incidence of postoperative complications among distal hypospadias was 23.4% (15.8–57.1%), and MAGPI, TIP, and Onlay procedures were mostly used, accounting for 15.3%, 69.4%, and 12.5%, respectively. The incidence of postoperative complications among middle hypospadias was 29.0% (22.7–40.0%), and TIP, Onlay, and Duckett procedures were mostly used, accounting for 52.3%, 20.6%, and 21.0%, respectively.

However, for the repair of proximal hypospadias, there is no surgical method that can achieve ideal results. In our multicenter study, 549 patients with proximal hypospadias were treated with Duckett method (59.2%), Koyanagi method (9.7%), and staged method (11.5%). However some physicians believe that staged surgery can obtain better function and appearance, and has a wide range of applications, thus it is being frequently chosen in these cases. Pippi Salle et al. [6] compared the three methods for treatment of proximal hypospadias and found that during the follow-up period of 30–48 months, the postoperative complication rate of long TIP was the highest at 53%, while the staged complication rate was the lowest at 32%. However, the postoperative complication rate of staged surgery was found to be higher with long-term follow-up. Stanasel et al. [7] reviewed 56 cases of patients with proximal hypospadias treated with staged Byars flap. During an average follow-up of 38.6 months, 68% of the patients developed complications that required further surgical intervention.McNamara et al. published long-term follow-up results of proximal hypospadias repaired initially by Retik staged surgery in 2015[8]. Among the 134 patients with a mean follow-up of 3.8 years and longest follow-up of 21.7 years, 53% developed complications after the second stage, and the reoperation rate was 49%. The most common complications were urethral fistula (29.1%), penile cleft (14.2%), and urethral stricture (12.7%). In our current study in China, complications were 30.8% (16/52) in TIP group, 38.5% (10/26) in Onlay group, 52.9% (172/325) in Duckett group, 40% in Duckett + Duplay group, and 30.2% in Koyanagi group. Complications of the staged operation group were 22.2%. There were statistically significant differences in postoperative complications among the six groups (*P* < 0.001), but because of the different penile conditions in each group of children, a certain surgical method cannot be considered as superior to other surgical methods. It is necessary to select the surgical method with the greatest long-term benefit according to penile condition and the experience of the individual operator.

In order to further reduce postoperative complications of hypospadias and select appropriate surgical methods for the different types, anatomical research data results on hypospadias from multiple centers should be analyzed at the present stage. This study focuses on TIP and Duckett techniques commonly used in China.

### Risk factors for short-term complications after TIP operation

In this multicenter study, the postoperative complication rate for TIP was 25% (84/336), including 53 cases of urethral fistula (15.8%), and 29 cases of urethral stricture (8.6%). Since the suture margin of the formed urethra and penis head are located on the ventral side of the penis, complications are more likely to occur after TIP when the glans is too small, and the UP is too narrow.

However, the effect of the width of the UP is still controversial. Nguyen et al. [9] studied the influence of UP characteristics on TIP and obtained the UP width range of < 8 mm or > 8 mm. There was no significant difference in postoperative complications of TIP with UP width < 8 mm or > 8 mm. However, Holland et al. [10] believed that UP width < 8 mm increased the incidence of urethral stricture after TIP. In our current study, UP width was defined the width of the UP by measuring widest distance between the lateral margins of the UP transversely. This multicenter study found that the width of the UP was 3–12 mm (mean 5 mm), and the width of the UP after longitudinal incision was 6–18 mm (mean 11 mm). Multivariate analysis found that the width of the UP after longitudinal incision (*OR* = 0.836, 95% *CI*: 0.742*–*0.942) was a risk factor for postoperative complications after TIP (*P* = 0.003), showing that each 1 mm increase in the width of the urethral plate after longitudinal incision reduced the risk of postoperative complications by 16.4%, while the width of the UP was not a risk factor for postoperative complications of TIP.

As for the glans width, some studies suggest that a penile head width of 14 mm in TIP represents the critical value, and the incidence of complications will be reduced by 20% when the glans size increases by 1 mm [11]. In this multicenter study, glans width was measured at the point of maximum width. Glans width was found to range from 7 to 25 mm with an average of 14.5 mm. Multivariate analysis indicated that glans width (*OR* = 0.851, 95% *CI*: 0.749*–*0.965) was an independent risk factor for postoperative complications after TIP surgery (*P* = 0.012), showing that the risk of postoperative complications was reduced by 14.9% for each 1 mm increase in glans width.

Bhat et al. [12] reported 14 cases of proximal hypospadias with well-developed UP and spongiform body accompanied by moderate penile curvature undergoing TIP operation. During the average follow-up of 30 months after surgery, two cases of urethral fistula and one case of urethral stricture occurred. Therefore, the characteristics of UP, such as flatness and elasticity, affect the therapeutic effect of TIP. In this study, the complications of the smooth UP group were less than those of the uneven UP groups (23.1% and 32.4%, respectively), and complications of the good UP elasticity group were less than those of the poor elasticity groups (23.3% and 31.4%, respectively), but the differences were not statistically significant. Among the children with proximal hypospadias treated with TIP, 88.5% had good UP elasticity, 84.6% had smooth UP, and the incidence of postoperative complications was 30.8%. Therefore, we suggest that when the elasticity and flatness of the UP are poor, TIP operation is not recommended, and transverse UP operation should be performed.

Snodgrass et al. [13] reported 58 cases of proximal hypospadias treated by TIP in 2021, 26% (15 cases) of which had recurrence of penis curvature during the average follow-up of 55 months after surgery. Among the cases, only dorsal plication was used in seven to correct penis curvature, and was done during the re-correction procedure. The ventral intact urethral plate leads to an increase in the tension of the ventral tissue of the penis, which ultimately leads to the recurrence of hypospadias. Therefore, for the surgical treatment of proximal hypospadias, Snodgrass suggests to only perform proximal TIP when there is little (< 30°) or no ventral curvature. In this study, only 9.5% of the children with proximal hypospadias were treated with TIP. The average penile curvature in the TIP group was 37.1°, and 44.2% had severe penile curvature. During the average follow-up of 26 months, no penile recurrence curvature occurred, which may be related to the strict understanding of the indications for TIP. Therefore, TIP should be used with caution for proximal hypospadias with severe penile curvature. Above all, for proximal hypospadias, when the flatness and elasticity of the UP are poor or when the penis is severely curved, TIP operation should be used with caution, and transverse urethral plate or staged operation should be selected.

### Risk factors of short-term complications after Duckett operation

Duckett operation is difficult and requires high surgical skills, with a high complication rate of 37.9–59.3% [14, 15]. Long et al. [16] reported that the incidence of complications in 167 cases of proximal hypospadias was 56% during the average follow-up of 31.7 months. Lucas et al. [17] found that postoperative complications of distal, intermediate, and proximal hypospadias were 10.7%, 18.8%, and 53.8%, respectively. In this multicenter study, the overall postoperative complication rate of distal hypospadias was 23.4%, that of middle hypospadias 29.0%, and that of proximal hypospadias 43.7%. Therefore, the postoperative complications of proximal hypospadias are high, which may be due to the length of urethral defect and the length of urethra to be formed.

Beijing Children's Hospital reported that urethral defect length (*OR* = 1.858, 95% *CI*: 1.053*–*3.277) was an independent risk factor for postoperative complications of Duckett surgery (*P* = 0.032) [18]. In this multicentrer study, the complication rate of Duckett operation was 51.2% (192/375), including 130 cases of urethral fistula (34.7%), 52 cases of urethral stricture (13.9%), and 54 cases of diverticular dilation (14.4%). Univariate analysis showed that there were no significant differences in age, penis length, glans length and width, and prepuce shape between the complication groups (*P* > 0.05). Univariate analysis showed that the length of urethral defect, length of prepuce island flap, and ventral curvature were the risk factors for complications after Duckett operation. Combined with clinical findings, we found that the above mentioned three factors are related to the severity of hypospadias. The more severe the degree of hypospadias, the longer the length of urethral defect after the correction, the longer the length of prepuce flap, and the greater the postoperative complications. At present, there are few studies on the effect of characteristics of prepuce island flaps on postoperative complications after Duckett procedure. The complications of smooth prepuce flaps were less than those of moderate and uneven flaps (42.0% < 71.1%), and those of good flap elasticity were less than those of general and poor flap elasticity (40.9% < 62.1%), and the differences were statistically significant (*P* < 0.001, *P* < 0.001). Combined with clinical practice, we believe that when the skin island flap is smooth and elastic, the incidence of postoperative complications may be relatively low. When the prepuce condition is poor, that is, when it is of uneven, and of poor elasticity, the inner wall of the newly formed urethra will not be smooth and will have poor elasticity, leading to a local vortex that forms during urination, resulting in the formation of urethral diverticulum.

However, multifactorial analysis in this study found that the elasticity (*P* = 0.195) and flatness of the skin island flap (*P* = 0.187), and ventral curvature(*P* = 0.594), were not independent risk factors for complications after Duckett operation, while the length of the prepuce island flap (*OR* = 3.506, 95% *CI*: 2.258*–*5.442) was an independent risk factor for postoperative complications after Duckett operation (*P* < 0.001). That is, the risk of postoperative complications increases 3.506× for each 1 cm increase in the length of the skin island flap. In conclusion, the length of skin island flap is considered to be a risk factor for postoperative complications after Duckett operation.

This multi-center clinical study has the advantage of analyzing a large number of cases. A total of 1011 cases of hypospadias with complete clinical and follow-up data were included in this study. However, the following problems still exist: due to regional environmental differences and unbalanced economic development in China, medical resources are not evenly distributed. This multi-center study only contains clinical data from provincial children's hospitals, which do not cover all children with hypospadias. It is difficult to control the quality of data from various units in a multi-center study, thus, it is necessary to further unify the measurement standards of various penis parameters and establish a standardized quality control system to ensure the authenticity and integrity of data.

## Conclusions

Several anatomical features play a role during the selection process among the different surgical approaches, including glans size, urethral plate width, and the meatal position.The width of UP and glans were risk factors for postoperative complications after TIP. The length of prepuce island flap was a risk factor for complications after Duckett operation.

## Data Availability

The datasets used and/or analysed during the current study available from the corresponding author (HongCheng Song and Ning Sun) on reasonable request.

## Abbreviations

UP: Urethral plate
MAGPI: Meatal advancement and glanuloplasty incorporated procedure
TIP: Tabularized incised plate urethroplasty
Duckett: Transverse preputial island flap urethroplasty
